# Case Report: an unusual case of a penetrating intracranial metallic foreign body removed via surgery

**DOI:** 10.3389/fsurg.2025.1588359

**Published:** 2025-04-30

**Authors:** Dang Tang, Hai Song, Bibo Gao, Jiang Long, Zhongkun Ren

**Affiliations:** Department of Neurosurgery, The First Affiliated Hospital of Kunming Medical University, Kunming, China

**Keywords:** intracranial foreign body, trauma, imaging, surgery, prognosis

## Abstract

**Background and importance:**

Intracranial foreign bodies resulting from criminal assaults that cause penetrating trauma are relatively rare. Such cases are often accompanied by significant complications, including intracranial hemorrhage, cerebral contusion, major vascular injury, and cerebrospinal fluid leakage, which pose substantial challenges in clinical management. Herein, we report a successful case of surgical treatment for an intracranial metallic foreign body, aiming to provide valuable insights for similar clinical scenarios.

**Clinical presentation:**

A 38-year-old male patient was urgently admitted to our emergency department following an intentional assault with a sickle, which resulted in an intracranial penetrating injury. Computed tomography (CT) scanning revealed the presence of a metallic foreign body that traversed the left temporal lobe and extended to the sphenoid bone and the posterior region of the right orbit. In response to this critical situation, an emergency surgical procedure was promptly initiated. The treatment strategy involved a combination of craniotomy and transnasal approaches to remove the metallic foreign body and reconstruct the skull base. Postoperatively, the patient exhibited no obvious adverse reactions, and his condition remained stable throughout the follow-up period.

**Conclusion:**

Intracranial foreign bodies often penetrate neural structures via the orbit or nasal cavity. When dealing with a long foreign body predominantly situated within the brain parenchyma, it is crucial to avoid blindly extracting it. Instead, shortening the length of the foreign body may be a more feasible approach to facilitate its safe removal and transportation. Preoperative acquisition of comprehensive imaging data is of utmost importance, as it aids in delineating the spatial relationship between the foreign body, major intracranial vessels, and cranial nerves, thereby enabling the formulation of a rational surgical plan. Whenever possible, the removal of intracranial foreign bodies should be carried out within 6–8 h post-trauma. Additionally, reliable skull base reconstruction is essential to prevent cerebrospinal fluid (CSF) leakage and mitigate the risk of infectious complications.

## Introduction

Traumatic intracranial foreign bodies are most commonly encountered in criminal activities or accidents. During wartime, they are often caused by military firearms or explosive fragments (1). Foreign bodies that penetrate the skull and brain can cause severe damage to the central nervous system. These foreign bodies can migrate spontaneously within the brain, leading to complications such as infection, epilepsy, vascular abnormalities, cerebrospinal fluid leakage, and mental disorders. However, there is limited literature on this topic, and specific treatment approaches and prognoses vary (2). Consequently, we present a case of an intracranial metallic foreign body managed using microscopical and endoscopic techniques. These techniques enable the removal of the foreign body without causing additional damage to adjacent intracranial tissues. The patient in this case had a satisfactory postoperative recovery.

## Case presentation

**Figure 1:**
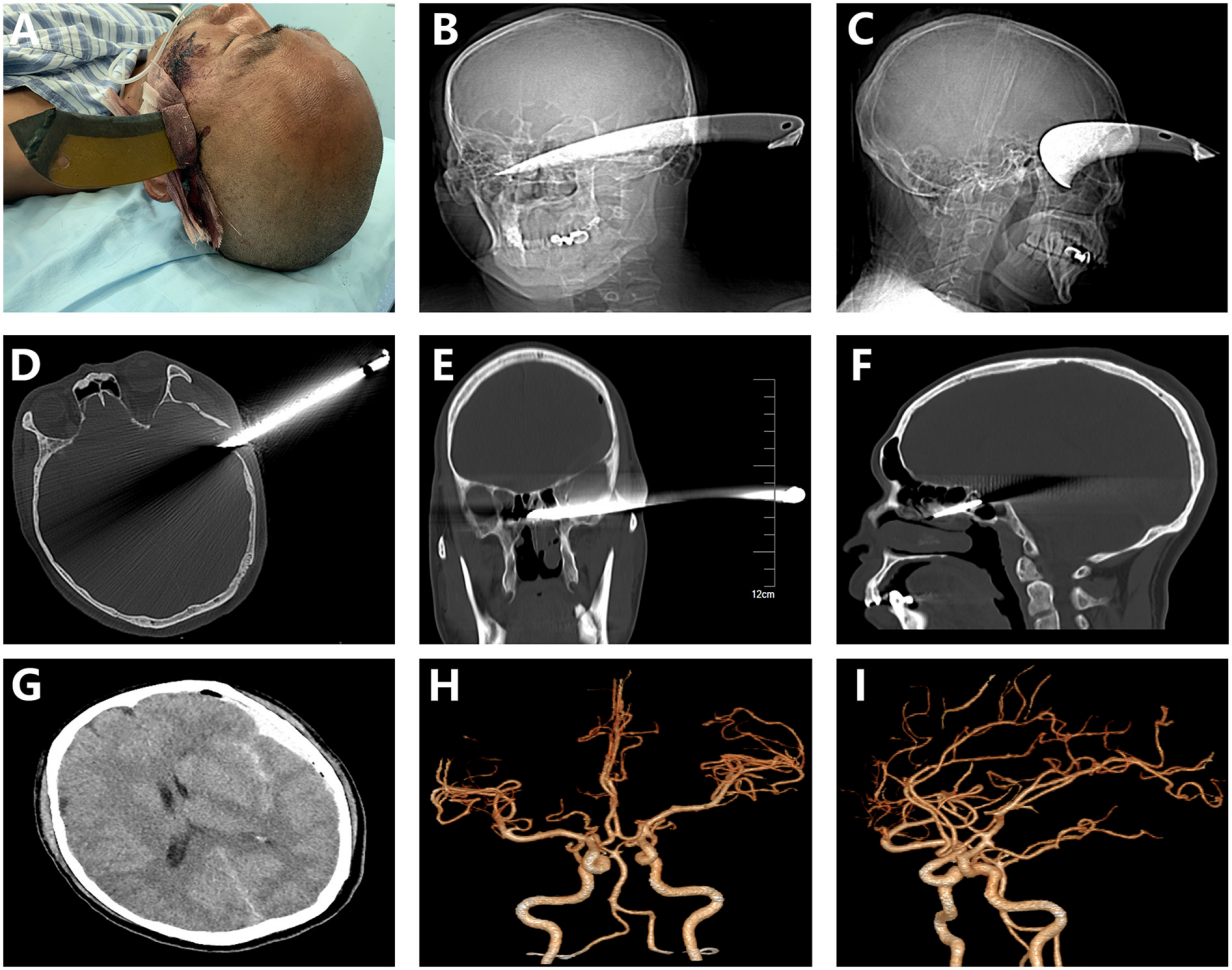
The preoperative image depicted a metallic foreign body that penetrated through the left temporal region **(A)**. Radiographic examination, along with computed tomography (CT) scans, clearly demonstrated that the metallic foreign body traversed the left temporal lobe, passed through the ethmoid and sphenoid sinuses, and extended to the sphenoid bone and the posterior part of the right orbit **(B–F)**. Axial CT imaging revealed the presence of subdural hematoma and subarachnoid hemorrhage **(G)**. Computed tomography angiography (CTA) findings indicated that there was no major injury to the intracranial arteries **(H,I)**.

**Figure 2:**
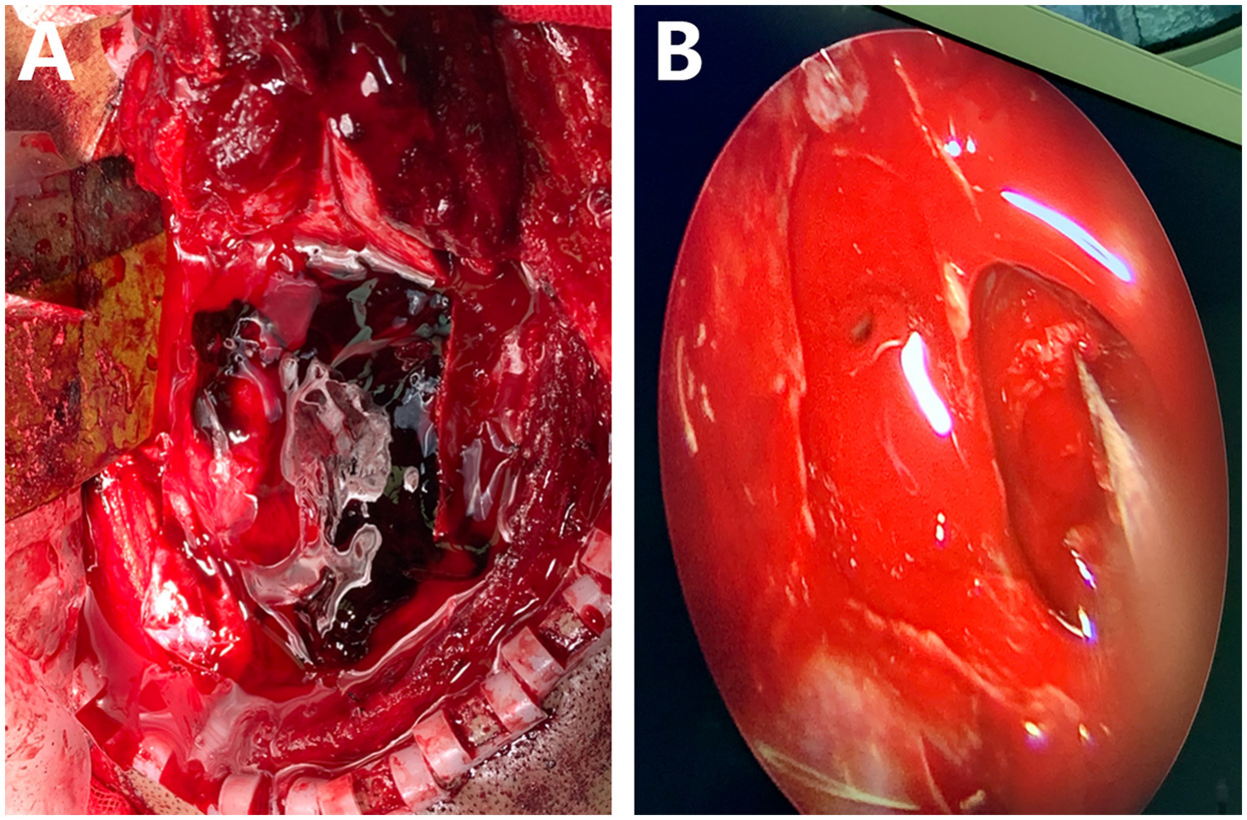
The intraoperative image showed that a metallic foreign body had penetrated through the brain tissue, resulting in the formation of a hematoma. The intracranial foreign body was completely exposed and was successfully removed under the microscope **(A)** Subsequently, endoscopic techniques were employed to repair the cerebrospinal fluid (CSF) leak and reconstruct the base of the skull **(B).**

**Figure 3:**
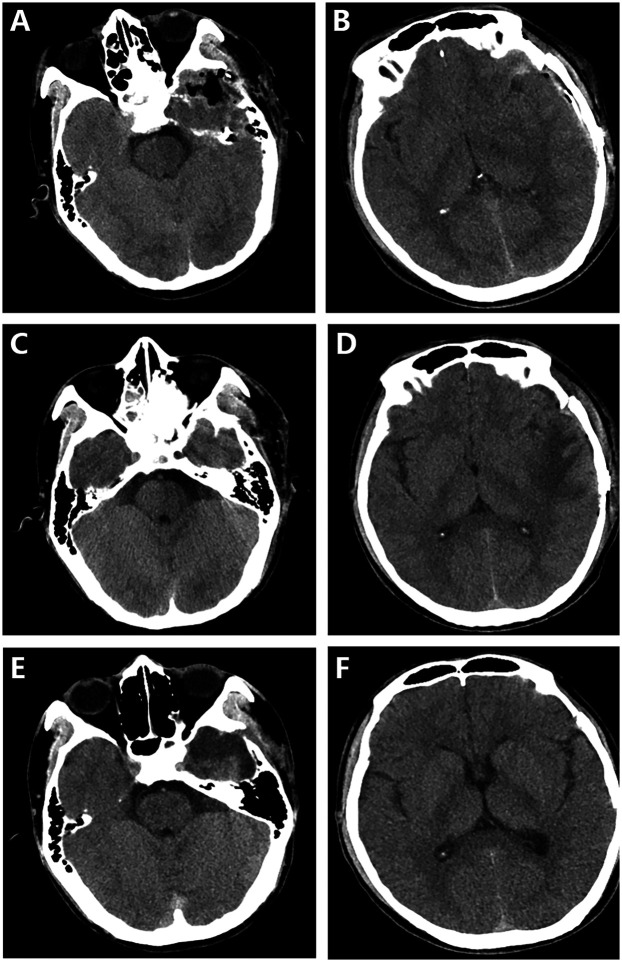
Postoperative computed tomography (CT) scans were conducted at different time points: 6 h **(A,B)**, 1 week **(C,D)**, and 1 month **(E,F)** after the surgery. The follow-up CT images clearly demonstrated the successful removal of both the foreign body and the hematoma, as well as satisfactory neurological recovery.

The patient, a 38-year-old man, was referred to the neurosurgery emergency department with a chief complaint of an intracranial foreign body remaining 5 h after an assault by others. Upon arrival at the emergency department, the fire brigade used a rod cutter to cut the part of the sickle outside the skull in the emergency room. The patient had no history of loss of consciousness following the injury. He was lethargic but oriented, with a Glasgow Coma Scale of 13 and stable vital signs (Temperature: 36.5°C, Pulse: 82 beats/min, Respiration: 18 breaths/min, Blood pressure: 140/85 mmHg, Blood oxygen saturation: 96%). Bilateral pupils were equal and reactive. Neurological examination was normal. The trauma was limited to the head, with no other visible injuries (Figure 1A). There was no history of any other illness or injury. Imaging data, including computed tomography (CT) and cerebral CT angiography, were promptly obtained after admission. The cerebral CT scan indicated a metallic foreign body passing through the left temporal lobe, traversing the ethmoid and sphenoid sinuses, and extending to the sphenoid bone and the posterior part of the right orbit, adjacent to the internal carotid artery. There was laceration in the left temporal lobe with subdural hematoma and subarachnoid hemorrhage (Figures 1B–I). Following the CT scan, an urgent surgery was performed using microscopical and endoscopic techniques (Figures 2A,B). Additionally, tetanus antitoxin, antibiotics, and antiepileptic drugs were administered. After the surgery, the patient was transferred to the neurosurgery intensive care unit (ICU). A postoperative cerebral CT examination showed no fresh intracranial bleeding and satisfactory reconstruction of the skull base (Figures 3A,B). Postoperatively, the patient had clear consciousness and stable vital signs, with no symptoms of neurological impairment or inflammation. No CSF leakage or intracranial infection occurred after the operation. Lumbar puncture on the fifth and eighth days after surgery showed no evidence of central nervous system infection. A postoperative cerebral CT scan 2 weeks later showed no purulent inclusions (Figures 3C,D). The patient was discharged from the hospital, and postoperative recovery was uneventful. He had regular follow-ups for 1 month without further complications (Figures 3E,F).

### Surgical procedure

The surgical objectives encompassed the removal of the intracranial foreign body, debridement of necrotic brain tissue, reconstruction of the anterior skull base, and prevention of CSF leakage. The principal challenge stemmed from the foreign body's close proximity to vital structures, namely the internal carotid artery and optic chiasma. The foreign body, crescent—shaped in morphology, measured 20 cm in length, 3 cm in width, and had a depth of 7 cm within the skull. It had penetrated deeply into the ethmoidal sinus, with its orientation perilously close to the internal carotid artery, cavernous sinus, and optic nerve, thereby rendering its removal arduous and mandating a craniotomy approach. Given the extensive injury to the skull base, appropriate reconstruction was essential for averting CSF leakage and postoperative infections.

Upon verification of the patient's information, endotracheal intubation was carried out, followed by the administration of general anesthesia. The skin incision was meticulously designed along the penetrating trajectory to optimally expose the underlying skull area. The dimensions and shape of the bone flap, measuring 5 × 6 cm, were determined based on the precise location and size of the foreign body. Post—craniotomy, the dura incision was carefully executed in accordance with the wound direction to afford unobstructed access to the foreign body. Under microscopic visualization, the hematoma and necrotic, contaminated brain tissue were removed and carefully separated from the foreign body. The foreign body was found to be adherent to the surrounding tissues, including the internal carotid artery, cavernous sinus, and optic nerve. Microsurgical techniques were thus employed to delicately dissect these adhesions. Subsequent to the removal of the foreign body, bipolar electrocautery and hemostatic agents were utilized to ensure the absence of active bleeding within the surgical field. The dura mater was then sutured closed, and the bone flap was securely fixed in place using titanium plates and screws.

A fronto—temporal craniotomy was performed to access the left middle skull base, with the pedicled periosteum conserved as the primary material for skull base repair. Under microscopic guidance, the left temporal lobe was gently elevated. After the removal of contusions and lacerations, the foreign body was fully visualized at the skull base, as corroborated by preoperative CT reconstruction. The foreign body was successfully removed following the careful loosening of the surrounding tissue. Concurrently, the necrotic and contaminated brain tissue in the vicinity of the foreign body was completely excised under microscopic magnification. During the removal of the foreign body, force was applied gradually in the direction opposite to the penetration. It is of utmost importance to note that the brain tissue should be meticulously separated along the periphery of the foreign body, ensuring its full exposure. Blind removal of the foreign body must be scrupulously avoided to prevent damage to large vessels and vulnerable nerve structures. The fractured middle skull base was repaired by comprehensively covering it with an appropriately sized muscle fascia and the pre—reserved pedicled periosteal flap. Neuroendoscopy, via a transnasal transseptal transsphenoidal approach, was employed to detect CSF leakage and precisely identify the leakage site. The dura was repaired using a nasal septal flap harvested from the septum. The overarching aim of CSF repair was to reconstruct the skull base, establish an effective barrier between the cranial cavity and the paranasal sinuses, and preserve the neurovascular integrity and function of the paranasal sinuses.

## Discussion

### Foreign body diagnosis

Foreign bodies mostly penetrate the brain through the facial skull, such as the superior orbital plate of the orbit, the medial orbital plate, the ethmoid plate of the nostril, and the facial area, as the bones in these parts are relatively fragile. For organic foreign bodies like wood, conventional CT and skull X-rays may yield negative results and be easily missed. Many patients may present with central nervous system symptoms such as throbbing headache, nausea, vomiting, and altered sensorium and seek medical treatment years later (3). Therefore, a detailed medical history should be obtained, and external wounds should be noted. Especially when non—metallic foreign bodies are suspected, MRI or ultrasound examination should be added if necessary. However, it must be ensured that there are no paramagnetic metal foreign bodies in the body before performing MRI.

### Whether to remove foreign bodies

When the foreign body is long and most of it is located outside the skull, and it cannot be loaded into the ambulance due to length constraints, it should not be blindly pulled out. Instead, its length can be shortened to facilitate transportation. If the foreign body has remained for a long time, is deeply located, there are no obvious neurological symptoms, seizures can be controlled by drugs, there is no sign of infection, and the surgical risk or the likelihood of postoperative neurological dysfunction is high, surgical removal of the foreign body is not recommended (4). However, it is generally acknowledged that organic foreign bodies such as wood have a high potential for bacterial growth (5). If the time since injury is short and simple wound exploration is sufficient to locate the foreign body, it can be removed while avoiding neurological deficits (6).

### Preoperative assessment

Rapid collection of imaging data before surgery is of utmost importance. Preoperative evaluation should determine the location of the foreign body and its relationship with blood vessels and important anatomical structures. For cases suspected of involving important blood vessels, three—dimensional reconstruction, CTA, or DSA should be performed to assess the relationship between the foreign body, blood vessels, and important nerve tissues and to determine the location and direction of the foreign body. If the foreign body is located deep within the brain, frameless stereotactic systems should be prepared preoperatively as much as possible, or intraoperative ultrasound can be used in combination. For patients with possible foreign body displacement, neurological dysfunction changes should be carefully observed, and CT should be reviewed in a timely manner to achieve real—time relocation before and during the operation.

### Surgery

Regarding the timing of surgery, if the patient's intracranial or systemic condition is stable, surgical treatment can be carried out after completing relevant examinations (7). Once surgery is decided, arrangements should be made as soon as possible to avoid secondary cerebral injury. There are three main methods for removing foreign bodies. First, if no major blood vessels are injured after assessment, the foreign body can be directly removed at risk. However, this operation carries the risk of massive bleeding, and corresponding craniotomy or intervention should be prepared, with timely postoperative CT review. Second, foreign bodies can be removed using stereotactic techniques. Burr hole surgery and two—dimensional biplanar image—guided stereotactic techniques with endoscopy can be used to remove thin but long foreign bodies lodged in deep brain tissue. Such special techniques are considered safe for elective removal of such objects (8). Third, the most common approach is craniotomy to remove foreign objects (9). When removing foreign bodies, the action should be gentle. Especially when the foreign body is embedded in the skull, efforts should be made to ensure the stability of the foreign body during craniotomy. During craniotomy, a small bone flap can be made immediately adjacent to the site where the foreign body enters the skull. If there is massive bleeding, the bone flap can be rapidly expanded. If the foreign body is difficult to remove, it should not be forced; instead, the foreign body should be exposed as much as possible, and the trapped tissue should be removed simultaneously before removing the foreign body. For multiple foreign bodies, intraoperative CT can be performed if necessary to determine whether all foreign bodies have been removed, avoiding a second operation. Debridement is an essential part of successful intracranial foreign body surgery and should be carried out as soon as possible. Complete debridement of the wound track is not always necessary; partial debridement to preserve neurological function does not significantly increase the risk of infection (10). The scope of debridement should be determined according to the intraoperative situation. The incidence of traumatic intracranial foreign body—related vascular injury is mainly related to the material of the foreign body, the injury mechanism, and the site of cranial entry. Common vascular injuries include traumatic aneurysm, arteriovenous fistula, venous sinus injury, and vascular occlusion (11). If large intracranial blood vessels are injured by foreign bodies, it is recommended to prepare sufficient blood before surgery, have two aspirators and aneurysm clips ready, and prepare for interventional treatment for rapid hemostasis.

### Infection prevention

As much as possible, the foreign body should be removed within 6–8 h after trauma, it implies that early treatment of wounds is crucial to prevent infection, which is relevant to the principle of timely removal of foreign bodies (12). During the operation, the dura should be repeatedly irrigated, and the dura should be repaired with a watertight closure. More importantly, reliable skull base reconstruction should be carried out to avoid cerebrospinal fluid leakage. Post—operation, anti—infection treatment with broad—spectrum antibiotics that can easily cross the blood—brain barrier should be administered. Generally, wound contamination with organic matter can lead to post—injury skin and soft tissue fungal infections, particularly mucormycosis. Wood is prone to infection due to its porous nature and tendency to fragment and can serve as a nutrient source for bacteria (13, 14). Some foreign bodies are chemically reactive; for example, certain metals like iron can corrode in the body's environment. The corrosion process can release metal ions, which may disrupt the normal physiological environment of the surrounding tissues, creating a more favorable environment for bacterial growth. Smooth—surfaced foreign bodies are less likely to harbor bacteria. Microorganisms have more difficulty adhering to smooth surfaces, and the flow of body fluids can more easily remove any bacteria that come into contact with the smooth foreign body. In contrast, rough—surfaced foreign bodies provide numerous niches and crevices where bacteria can attach, multiply, and form biofilms (15). Once a biofilm is formed, it becomes more difficult for the immune system and antibiotics to reach the bacteria, significantly increasing the risk of persistent infections. The infection rate is relatively high in patients with foreign bodies passing through the paranasal sinus and orbital regions. Intracranial infection is associated with a high incidence of penetrating head trauma (16). The frontal, ethmoid, and sphenoid sinuses are in close proximity to the anterior and middle cranial fossae. If a penetrating intracranial trauma occurs in the frontal bone or ethmoid area, there is a high risk of introducing bacteria from the sinuses into the intracranial cavity. A fracture or penetration through the thin bony walls separating the sinuses from the brain can create a pathway for these organisms to enter the intracranial space, leading to meningitis, brain abscesses, or subdural empyemas. According to our experience, in cases of definite infection, the anti—infection treatment course is more than 2 weeks. If there is no specific infection, the prophylactic antibiotic regimen, consisting of 1 g of cefazolin every 8 h, is administered for 5–7 days (17).

### Epilepsy

The occurrence of epilepsy in patients with traumatic intracranial foreign bodies is closely related to primary brain injury and foreign body retention. Epilepsy and status epilepticus are common symptoms when intracranial foreign bodies have been present for a long time, and sudden—onset seizures are typical symptoms of both recent and concealed penetrating brain injuries (18). Late—onset seizures can be caused by slow gliosis, progressive granulomatous changes, prolonged abscess formation, and metal toxicity in cases of retained foreign bodies (19). The incidence of epilepsy in patients with penetrating brain injury is approximately 30%—50% (20). This patient did not experience seizures after surgery and was followed up for 1 month. Currently, there is insufficient evidence—based medical evidence for the preventive use of antiepileptic drugs, so patients with minor brain damage do not need to use them. In addition, the preventive use of antiepileptic drugs should not exceed 7 days. After about 7 days, the risk of seizures due to acute injury typically decreases. Limiting the preventive use to 7 days helps minimize exposure to potential adverse effects while still providing a window of protection against seizures likely to occur in the acute phase (21, 22).

## Conclusion

In summary, the surgical principle for intracranial foreign bodies is mainly debridement, including removing foreign bodies, fragmented brain tissue, bone fragments, and hematoma, relieving intracranial hypertension, and repairing dural and scalp defects, thereby converting the open and contaminated wound into a closed and clean one. Surgery should be performed as soon as possible. The earlier the operation, the faster the wound heals, and the lower the infection rate, ultimately reducing long—term complications and adverse reactions. The management of brain foreign bodies depends on the location and injury pattern of penetrating injuries, as well as the composition of the object, to determine the treatment strategy. As this is a single—case study, the conclusions drawn are specific to this particular case, and further research involving larger cohorts is necessary to establish more generalized clinical guidelines.

## Data Availability

The datasets presented in this article are not readily available because of ethical and privacy restrictions. Requests to access the datasets should be directed to the corresponding author.
