# Supplementary figures and images for: Case Report: an unusual case of a penetrating intracranial metallic foreign body removed via surgery
Source: Front Surg. 2025 Apr 30;12:1588359. doi: 10.3389/fsurg.2025.1588359 (PMC12075331; doi:10.3389/fsurg.2025.1588359)

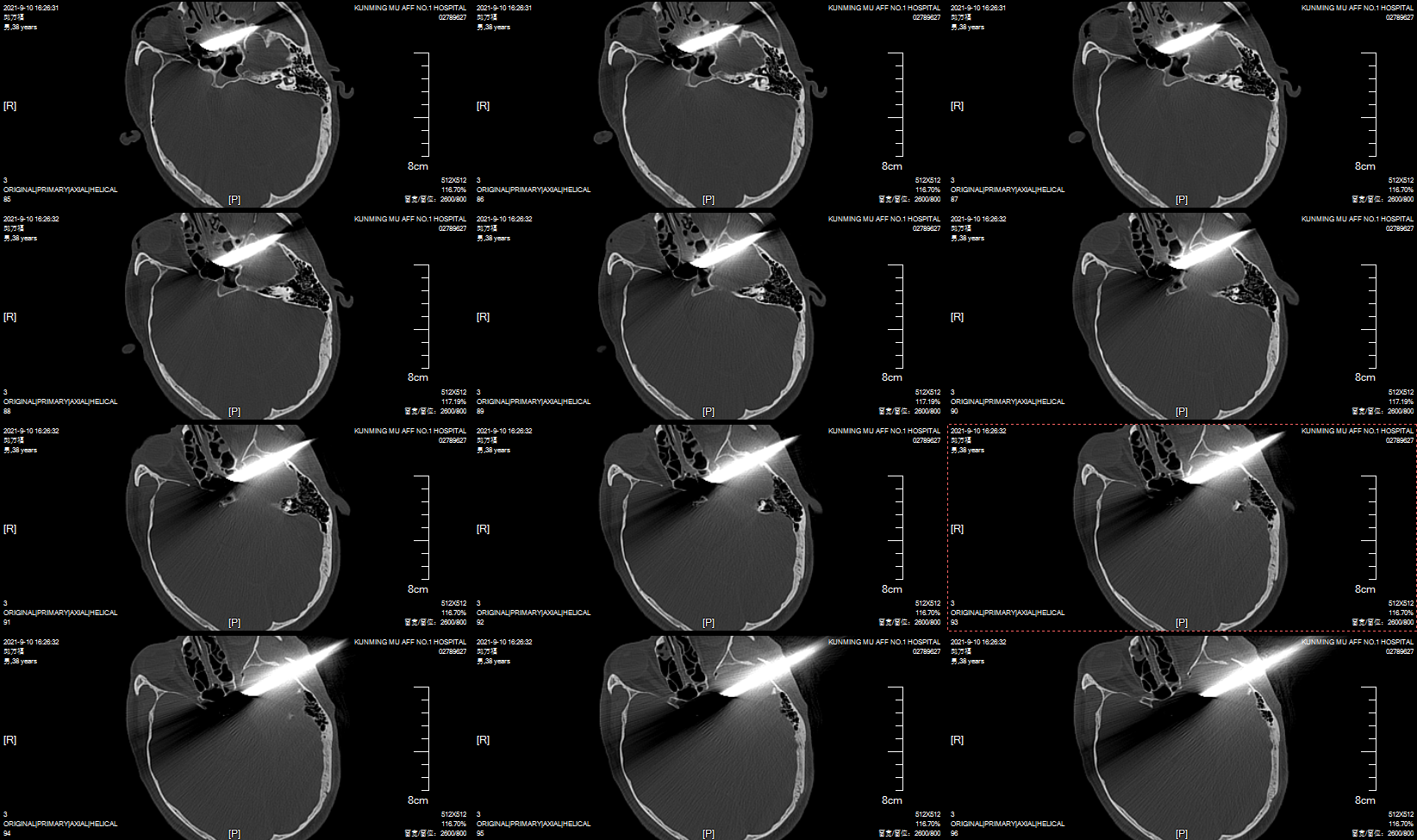

Supplement: Supplementary file 1 [file Image1.png]

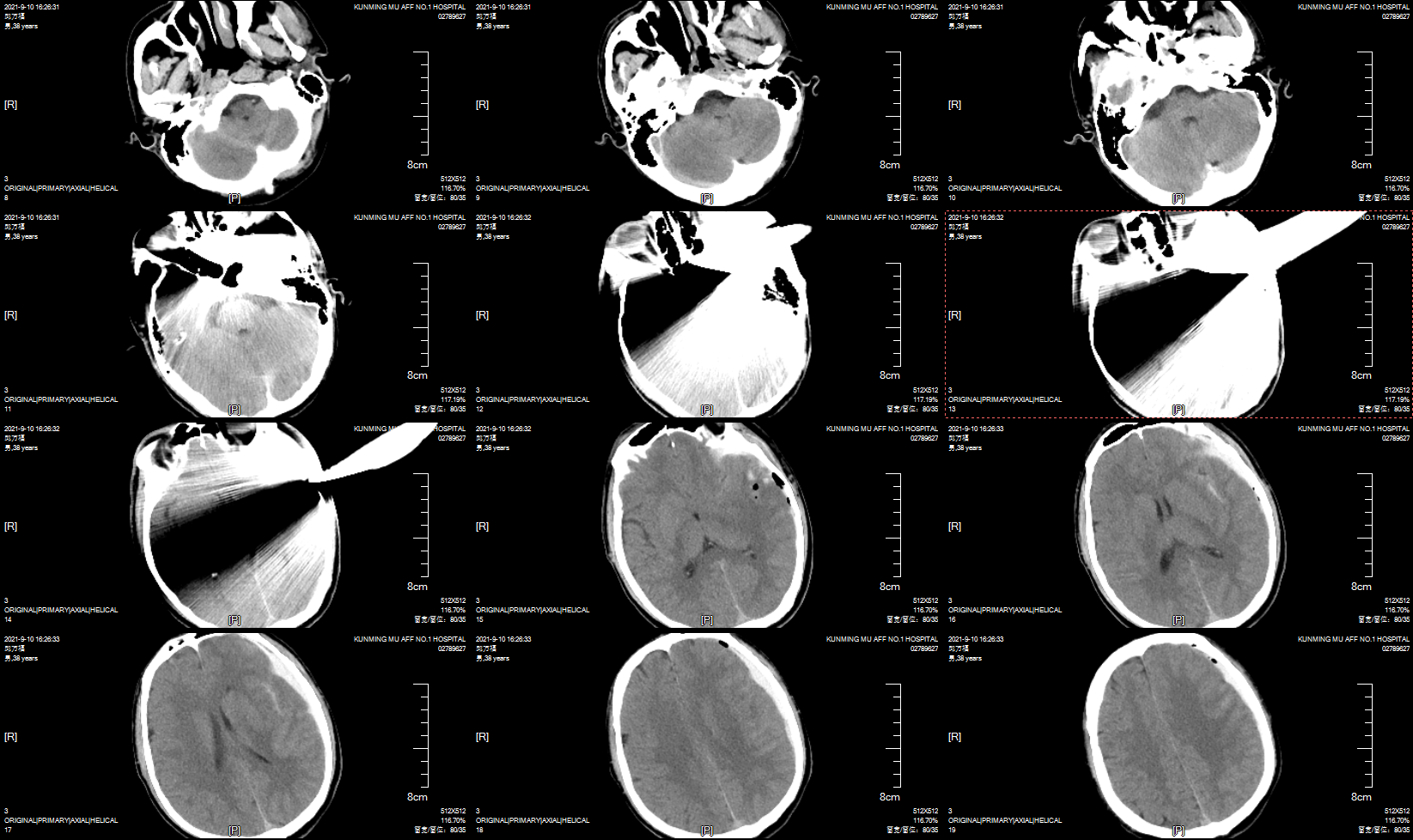

Supplement: Supplementary file 2 [file Image2.png]
