# Supplementary material for: Case Report: an unusual case of a penetrating intracranial metallic foreign body removed via surgery
Source: Front Surg. 2025 Apr 30;12:1588359. doi: 10.3389/fsurg.2025.1588359 (PMC12075331; doi:10.3389/fsurg.2025.1588359)
Supplement: Supplementary file 4 [file Table2.docx]

Dear reviewer,

We appreciate your careful review and insightful suggestion. According to your advice, we industriously amended the relevant part in manuscript step by step.

1. The authors make many statements, oftentimes vague, without providing references, for example:
- “If the foreign body remains for a long time, the location is deep, there are no obvious neurological symptoms, the seizure can be controlled by drugs, there is no sign of infection, and the risk of surgery or the possibility of postoperative neurological dysfunction is high, it is not recommended to remove the foreign body by surgery.” (see “whether to remove foreign bodies”)

Del Verme J, Giordan E, Marton E, Zanata R, Di Paola F, Canova G, Longatti P. Classification of orbitocranial wooden foreign body penetration injuries: what to do when they violate the intracranial space? A systematic review. J Neurosurg Sci. 2020 Apr;64(2):190-199. doi: 10.23736/S0390-5616.19.04793-3. Epub 2019 Nov 13. PMID: 31738026.

- “Complete debridement is not necessary, and partial debridement that preserve brain function does not significantly increase the chance of infection” (see subsection “surgery”)

Winder MJ, Monteith SJ, Lightfoot N, Mee E. Penetrating head injury from nailguns: a case series from New Zealand. J Clin Neurosci. 2008 Jan;15(1):18-25. doi: 10.1016/j.jocn.2007.06.020. Epub 2007 Nov 26. PMID: 18032048.

- “If the time after injury is not long and the location is shallow, the foreign bodies can be taken out on the basis of not aggravating nerve function injury.” (see subsection “whether to remove foreign bodies”)

Skinner EJ, Morrison CA. Wound Foreign Body Removal. 2023 May 1. In: StatPearls [Internet]. Treasure Island (FL): StatPearls Publishing; 2025 Jan–. PMID: 32119334.

- “Regarding the timing of surgery, if the patient’s intracranial or systemic condition is still stable, the surgical treatment can be performed after completing relevant examinations.” (see subsection “surgery”)

Campbell EA, Wilbert CD. Foreign Body Imaging. 2023 Jul 30. In: StatPearls [Internet]. Treasure Island (FL): StatPearls Publishing; 2025 Jan–. PMID: 29262105.

- «As far as possible, the foreign body should be removed within 6-8 hours after the dura…” (see subsection “infection prevention”)

The foreign body was removed as early as possible because a delay would have increased the possibility of infection or formation of a wrapped membranous structure, which would have in turn increased the difficulty of surgical removal.

Liu Q, Liu F, Xie H, Dong J, Chen H, Yao L. Emergency Removal of Ingested Foreign Bodies in 586 Adults at a Single Hospital in China According to the European Society of Gastrointestinal Endoscopy (ESGE) Recommendations: A 10-Year Retrospective Study. Med Sci Monit. 2022 Jul 26;28:e936463. doi: 10.12659/MSM.936463. PMID: 35879885; PMCID: PMC9339224.

- “Debridement is an indispensable part of successful intracranial foreign body operation and should be carried out as soon as possible. Complete debridement is not necessary, and partial debridement that preserve brain function does not significantly increase the chance of infection.” (see subsection “surgery”)
- “As far as possible, the foreign body should be removed within 6-8 hours after the trauma…” (see subsection “infection prevention”)

Liu Q, Liu F, Xie H, Dong J, Chen H, Yao L. Emergency Removal of Ingested Foreign Bodies in 586 Adults at a Single Hospital in China According to the European Society of Gastrointestinal Endoscopy (ESGE) Recommendations: A 10-Year Retrospective Study. Med Sci Monit. 2022 Jul 26;28:e936463. doi: 10.12659/MSM.936463. PMID: 35879885; PMCID: PMC9339224.

- “Generally speaking, the infection rate increases when the foreign matter is wood and other organic matter.” (see subsection “infection prevention”)

Generally speaking, wound contamination with organic matter can lead to post-disaster skin and soft tissue fungal infections, notably mucormycosis.

Wood is prone to infection including its porous quality and predisposition to fragmentation.

[1]Benedict K, Park BJ. Invasive fungal infections after natural disasters. Emerg Infect Dis. 2014 Mar;20(3):349-55. doi: 10.3201/eid2003.131230. PMID: 24565446; PMCID: PMC3944874.

[2] Miller CF, Brodkey JS, Colombi BJ. The danger of intracranial wood. Surg Neurol. 1977 Feb;7(2):95-103. PMID: 835079.

[3] Wieland AM, Curry WT, Durand ML, Holbrook EH. Management of a long-standing organic intracranial foreign body. Skull Base. 2010 Nov;20(6):487-90. doi: 10.1055/s-0030-1261264. PMID: 21772810; PMCID: PMC3134818.

In the introduction, the authors make a vague comparison with an unknown clinical picture. It remains unclear what the authors are trying to express:
- “However, there is less literature available on this subject and the specific treatment and prognosis are also different.” (see “Introduction”)

Voss JO, Thieme N, Doll C, Hartwig S, Adolphs N, Heiland M, Raguse JD. Penetrating Foreign Bodies in Head and Neck Trauma: A Surgical Challenge. Craniomaxillofac Trauma Reconstr. 2018 Sep;11(3):172-182. doi: 10.1055/s-0038-1642035. Epub 2018 Apr 24. PMID: 30087746; PMCID: PMC6078685.

2. The authors vaguely describe a microscopic removal of the sickle. Unfortunately, no details are given regarding the size of the intracranial portion, but it is hardly comprehensible that a sickle is removed microscopically. Attention should be paid to the correct choice of words here:

- “Consequently, we present a case of intracranial metallic foreign body removal by microscopical and endoscopic technique.” (see “Introduction”)

Consequently, we present a case of intracranial metallic foreign body managed by microscopical and endoscopic technique.

Corticectomy was microscopically performed overlying the metal blade. When removing the foreign body, the force was applied slowly against the direction of penetration.

- “The foreign body was too large, which was difficult to remove directly, and it needed to be removed by craniotomy.” (see “Surgical procedure”) (> what does too large mean?)

The foreign body was penetrating deeply (7 cm inside the skull).

3. There are several more examples where the choice of words is incorrect and leads to senseless sentences:

- “Imaging data including computed tomography (CT) and cerebral CT angiography were quickly improved after admission.” (see “Case presentation”) (> how do you improve a CT?)

performed

- “Following the CT scan, an urgent neurosurgical intervention was performed by microscopical and endoscopic technique.” (see “Case presentation”) (> what neurosurgical intervention was performed?)

an urgent surgery

- “… and there were no symptoms of neurological impairment and irritation.” (see “Case presentation”) (> what is a neurological irritation?)

There were no clinical or laboratory signs of inﬂammation

- “After conforming the information, endotracheal intubation and general anesthesia was performed.” (see “surgical procedure”) (> what information was confirmed?)

The information of the patient

- “If the foreign body is located in the deep location of the brain, the preoperative navigation preparation should be improved as much as possible…” (see subsection “preoperative assessment”) (> how do you improve the preoperative navigation preparation and what is this?)

The preoperative frameless stereotactic systems preparation should be acquired as much as possible.

Navigation refers to frameless stereotactic systems and facilitates localization of deep-seated foreign bodies.

4. In the description of the surgery technique, the authors describe a frontotemporal craniotomy using a left middle skull base approach. However, it must be assumed that a fronto-temporal craniotomy was performed first to gain access to the skull base:

- “Frontotemporal craniotomy via left middle skull base approach, …” (see “surgical procedre")

Fronto-temporal craniotomy was performed to gain access to the left middle skull base.

5. The authors describe a case of a sickle injury in which the anterior part of the sickle remains stuck in the skull and thus intracranially. In the discussion, however, it is described that foreign bodies can be removed “stereotactically” (see subsection “surgery”) or even left intracranially (see subsection “how to remove foreign bodies”).

There are three main ways to remove foreign bodies including stereotactic technique.

Muhammad AK, Maruno M, Maeda N, Kato A, Yoshimine T. Syringe needle located deep in the brain: image-guided removal. Surg Neurol. 2000 Dec;54(6):458-63; discussion 463-4. doi: 10.1016/s0090-3019(00)00311-6. PMID: 11240179.

The size ratio of a sickle that pierces bone and parenchyma over a long distance does not seem to have been taken into consideration here.:

The size ratio of a sickle is 7×3 cm inside the skull.


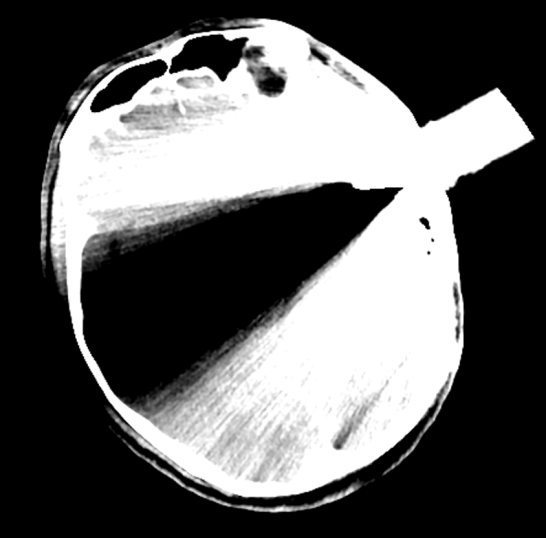


- “If the foreign body remains for a long time, the location is deep, there are no obvious neurological symptoms, the seizure can be controlled by drugs, there is no sign of infection, and the risk of surgery or the possibility of postoperative neurological dysfunction is high, it is not recommended to remove the foreign body by surgery.” (see “whether to remove foreign bodies”)

It is not recommended to remove the foreign body by surgery, if it is above situation.

Del Verme J, Giordan E, Marton E, Zanata R, Di Paola F, Canova G, Longatti P. Classification of orbitocranial wooden foreign body penetration injuries: what to do when they violate the intracranial space? A systematic review. J Neurosurg Sci. 2020 Apr;64(2):190-199. doi: 10.23736/S0390-5616.19.04793-3. Epub 2019 Nov 13. PMID: 31738026.

6. The authors describe vague experience regarding the duration of antibiotic therapy without substantiating this with concrete data or providing actual treatment suggestions:

- “According to our experience, in the case of definite infection, the anti-infection course of treatment is more than 2 weeks, and if there’s no specific infection, the course of treatment is 5 to 7 days.” (see subsection “infection prevention”)

If there is no specific infection, the course of administration of prophylactic antibiotics consisting of 1gm of cefazolin every 8h is 5 to 7 days.

Ganga A, Leary OP, Sastry RA, Asaad WF, Svokos KA, Oyelese AA, Mermel LA. Antibiotic prophylaxis in penetrating traumatic brain injury: analysis of a single-center series and systematic review of the literature. Acta Neurochir (Wien). 2023 Feb;165(2):303-313. doi: 10.1007/s00701-022-05432-2. Epub 2022 Dec 19. PMID: 36529784; PMCID: PMC9922212.

7. There are some incomplete or inconclusive sentences in the manuscript. The script appears to be unedited and unfinished:

- “The patient without any complications after the surgery, and postoperative result was favorable.” (see “Abstract”)

After the surgery, no obvious adverse reactions were found and the patient felt well during the follow-up.

- “With great size of foreign body in the brain conspired with poor prognosis.” (see “Introduction”)

Foreign bodies penetrating the skull and brain can cause serious central nervous system damage.

- “Many patients are due to secondary infections, epilepsy, etc. after injury to seek medical treatment in several years.” (see subsection “foreign body diagnosis”)

Many patients may be associated central nervous system symptoms such as throbbing headache, nausea, vomiting, and altered sensorium to seek medical treatment in several years.

- “At present, lack of evidence-based medical evidence for preventive use of antiepileptic drugs, so patients with minor brain damage do not need to use, and preventive use of antiepileptic drugs should not exceed 7 days.” (see subsection “epilepsy”)

At present, evidence-based medical evidence for preventive use of antiepileptic drugs is deficient, so patients with minor brain damage do not need to use. In addition, preventive use of antiepileptic drugs should not exceed 7 days.

Thank you very much for your constructive suggestion and comments. I have revised the text accordingly. Please check if we need any other changes or not.
