# Supplementary material for: Case Report: an unusual case of a penetrating intracranial metallic foreign body removed via surgery
Source: Front Surg. 2025 Apr 30;12:1588359. doi: 10.3389/fsurg.2025.1588359 (PMC12075331; doi:10.3389/fsurg.2025.1588359)
Supplement: Supplementary file 5 [file Table3.docx]

Dear reviewer,

We appreciate your careful review and insightful suggestion. According to your advice, we make every effort to amend the relevant part in manuscript step by step.

English language revision: The English used contains grammatical, syntactical, and terminological errors that compromise the clarity and readability of the text. A thorough review is necessary to enhance the linguistic and scientific quality of the article.

Thanks very much for your comments. We have reviewed and revised the entire article. Please see if the revised version met the English presentation standard.

Insufficient technical depth: The description of technical challenges encountered during surgery, such as managing the proximity to vital structures, is lacking. Including details on strategies adopted to minimize risks would have enriched the analysis.

Skin incision was designed along the penetrating direction to expose the underlying skull area, and the size and shape of the bone flap (5×6cm) were determined by the location and size of the foreign body. After the craniotomy, the dura incision was carefully made in accordance with the direction of the wound to provide optimal access to the foreign body. The hematoma and necrotic contaminated brain tissue were removed and separated from the foreign body under microscopic vision. The foreign body was adherent to the surrounding tissues including internal carotid artery, cavernous sinus and optic nerve, using microsurgical techniques to carefully dissect the adhesion. After removing the foreign body, using bipolar electrocautery and hemostatic agents to ensure that there is no active bleeding in the surgical field. Close the dura mater with sutures and fix the bone flap in place using titanium plates and screws.

Incomplete information on the foreign body: The dimensions, shape, and orientation of the foreign body are inadequately specified, details that are fundamental to understanding the complexity of the case.

The foreign body was shaped like a crescent moon, and the dimension of its length was 20cm, width was 3cm, and depth was 7cm inside the skull. The foreign body was penetrating deeply into the ethmoidal sinus, and its orientation was close to internal carotid artery, cavernous sinus and optic nerve.

Limited discussion of surgical alternatives: The article does not explore other operative options, such as minimally invasive techniques, nor does it provide a comparison with similar cases.

Burr hole surgery and the two-dimensional biplanar image-guided stereotactic technique with endoscopy were performed to remove the thin but long foreign body lodged in the deep brain tissue. The special technique would regard as the safe, elective removal of such objects.

Epilepsy prevention: The rationale for administering antiepileptic drugs is not adequately discussed in the context of guidelines or scientific evidence.

Epilepsy and status epilepticus are the common symptoms when intracranial objects account for the long existence, as well as sudden-onset seizures are typical symptoms of both recent and concealed intracranial penetrating injuries.

Late-onset seizures can be due to slow gliosis, progressive granulomatous changes, prolonged abscess formation, and metal toxicity in cases of retained foreign bodies.

Prolonged use of antiepileptic drugs (AEDs) is associated with an increased risk of side effects. These can include drowsiness, dizziness, nausea, and in more severe cases, liver and blood cell abnormalities. Limiting the preventive use to 7 days helps to minimize the exposure to these potential adverse effects while still providing a window of protection against seizures that are likely to occur in the acute phase. However, after about 7 days, the risk of seizures due to the acute injury typically decreases, and the benefit of continued preventive AED use may be outweighed by the risks.

Infection management: The discussion on infection risk could be expanded to analyze the relationship between the type of foreign body, trauma location, and the risk of infectious complications.

Generally speaking, wound contamination with organic matter can lead to post-disaster skin and soft tissue fungal infections, notably mucormycosis. Wood is prone to infection including its porous quality and predisposition to fragmentation, and it can serve as a nutrient source for bacteria.

Some foreign bodies are chemically reactive. For example, certain metals like iron can corrode in the body's environment. The corrosion process can release metal ions, which may disrupt the normal physiological environment of the surrounding tissues. This disruption can create a more favorable environment for bacteria to grow. Smooth - surfaced foreign bodies are less likely to harbor bacteria. Microorganisms have a harder time adhering to smooth surfaces, and the flow of body fluids can more easily remove any bacteria that come into contact with the smooth foreign body. On the other hand, rough - surfaced foreign bodies provide numerous niches and crevices where bacteria can attach, multiply, and form biofilms. Once a biofilm is established, it becomes more difficult for the immune system and antibiotics to reach the bacteria, significantly increasing the risk of persistent infections.

The frontal, ethmoid, and sphenoid sinuses are in close proximity to the anterior and middle cranial fossae. If a penetrating intracranial trauma occurs in the regions of the frontal bone or the ethmoid area, there is a high risk of introducing bacteria from the sinuses into the intracranial cavity. A fracture or penetration through the thin bony walls separating the sinuses from the brain can create a pathway for these organisms to enter the intracranial space, leading to meningitis, brain abscesses, or subdural empyemas.
