# Supplementary material for: Case Report: an unusual case of a penetrating intracranial metallic foreign body removed via surgery
Source: Front Surg. 2025 Apr 30;12:1588359. doi: 10.3389/fsurg.2025.1588359 (PMC12075331; doi:10.3389/fsurg.2025.1588359)
Supplement: Supplementary file 3 [file Table1.docx]

Subject: Resubmission of Manuscript An Unusual Case of a Penetrating Intracranial Metallic Foreign Body Removed via Surgery with Revisions

Dear Professor,

I hope this email finds you well. We are writing to submit the revised version of our manuscript titled “An Unusual Case of a Penetrating Intracranial Metallic Foreign Body Removed via Surgery” for your kind consideration for publication in Frontiers in Neurology. Because there are major problems related to the language. Our manuscript was previously reviewed and unfortunately rejected, and we sincerely appreciate the time and effort that you and the reviewers dedicated to evaluating our work.

In response to this feedback, we have taken extensive and meticulous steps to enhance the scientific quality and rigor of our manuscript.

Enhanced the writing style: We have carefully edited the manuscript to improve the clarity and readability of the text, and have ensured that the language is consistent throughout the manuscript.

We believe that these revisions have significantly improved the quality of our manuscript and have addressed all the concerns raised by the reviewers. We are confident that our study makes a valuable contribution to the field of neurosurgery and that it will be of interest to the readers of Frontiers in Neurology.

We would like to take this opportunity to thank you again for your time and consideration. If you have any further questions or require additional information, please do not hesitate to contact us.

Sincerely,

Dang Tang

Department of Neurosurgery, The First Affiliated Hospital of Kunming Medical University, Kunming, Yunnan 650101, China

March 5th, 2025
